# Psychological Balance in High Level Athletes: Gender-Based Differences and Sport-Specific Patterns

**DOI:** 10.1371/journal.pone.0019007

**Published:** 2011-05-04

**Authors:** Karine Schaal, Muriel Tafflet, Hala Nassif, Valérie Thibault, Capucine Pichard, Mathieu Alcotte, Thibaut Guillet, Nour El Helou, Geoffroy Berthelot, Serge Simon, Jean-François Toussaint

**Affiliations:** 1 Institut de Recherche Bio-Médicale et Epidémiologique du Sport (IRMES), Institut National du Sport, de l'Expertise et de la Performance (INSEP), Paris, France; 2 Institut National de la Santé et de la Recherche Médicale(INSERM), Unit 970, Paris, France; 3 Université Paris-Descartes, Paris, France; 4 Centre d'Accompagnement et de Prévention pour les Sportifs (CAPS), Centre Hospitalier Universitaire, Bordeaux, France; 5 Département Médical, Institut National du Sport, de l'Expertise et de la Performance (INSEP) Paris, France; 6 Centre d'Investigations en Médecine du Sport (CIMS), Hôtel-Dieu, Assistance Publique des Hôpitaux de Paris, Paris, France; University of Michigan, United States of America

## Abstract

**Objectives:**

Few epidemiological studies have focused on the psychological health of high level athletes. This study aimed to identify the principal psychological problems encountered within French high level athletes, and the variations in their prevalence based on sex and the sport practiced.

**Methods:**

Multivariate analyses were conducted on nationwide data obtained from the athletes' yearly psychological evaluations.

**Results:**

A representative sample of 13% of the French athlete population was obtained. 17% of athletes have at least one ongoing or recent disorder, generalized anxiety disorder (GAD) being the most prevalent (6%), followed by non-specific eating disorders (4.2%). Overall, 20.2% of women had at least one psychopathology, against 15.1% in men. This female predominance applied to anxiety and eating disorders, depression, sleep problems and self-harming behaviors. The highest rates of GAD appeared in aesthetic sports (16.7% vs. 6.8% in other sports for men and 38.9% vs. 10.3% for women); the lowest prevalence was found in high risk sports athletes (3.0% vs. 3.5%). Eating disorders are most common among women in racing sports (14% vs. 9%), but for men were found mostly in combat sports (7% vs. 4.8%).

**Discussion:**

This study highlights important differences in psychopathology between male and female athletes, demonstrating that the many sex-based differences reported in the general population apply to elite athletes. While the prevalence of psychological problems is no higher than in the general population, the variations in psychopathology in different sports suggest that specific constraints could influence the development of some disorders.

## Introduction

With the development of applied sports psychology research over the past decades, increasing attention has been turned to the psychological well-being of elite athletes, who not only make colossal mental and physical investments into their sport, but also deal with tremendous pressures in order to succeed at the international level. For many, sports participation represents a recreational and social outlet as well as an effective method to cope with stress, and is well known to prevent the onset of many psychological problems such as depression [1]–[4] or anxiety disorders [2], [4]. However, practicing a sport at the highest level offers an entirely different set of circumstances, representing a high pressure career replete with stressors and constraints. Some of the psychological issues encountered at the elite level and within particular sports have been studied in depth, and are sometimes highly mediatised, such as depression and anxiety stemming from season or career-ending injuries [5], [6], disordered eating in women involved in weight-bearing or aesthetic sports [7], [8] or the abuse of illegal performance-enhancing substances [9], [10]. However, data on the overall psychological health of elite athletes is difficult to obtain at an epidemiological level, and the athletic populations studied instead are often high school or college sportsmen and women-most of whom never reach the high level. Findings from these studies may be difficult to apply to the elite athlete population, who, by a favorable combination of genetic predispositions, personality and environmental circumstances [11], [12] added to relentless motivation and hard work, have been able to rise to the top in their sport. While the psychological resilience attributed to high level athletes [13] aids their ascension to the world stage and allows them to succeed where others are eliminated from the competition, this mental toughness does not imply complete immunity from psychopathology. Rather, even in relatively resilient individuals, provided enough adverse circumstances are met, it is conceivable that the added pressures inherent to the high level could facilitate the development of psychological problems.

With the increased participation of women in high level sport, the study of gender differences in many facets of sport and exercise has bloomed as a field of research. While a large body of literature exists on the gender differences in psychopathology for the general population, such large scale psychological data on male and female elite athletes is scarce. The most striking gender disparities in psychopathology are preserved across populations, of different ages or income levels. Women are diagnosed with depression and anxiety disorders roughly twice as often as men [14]–[16] and suffer from eating disorders 6 to 10 times more often than men [17] , while a notable male preponderance exists in alcohol abuse and dependence and externalizing disorders, such as alcohol and drug abuse [14], [18], [19]. It would therefore be reasonable to expect that even among elite athletes, these gender-based disparities would be observed.

To our knowledge, no published study has yet gathered data on the global psychological status of a large, nationally-representative sample of high-level athletes, from a wide range of Olympic sports, encompassing the most common psychological disorders as well as the social and personal factors likely to influence their occurrence. This study aims to bring forth the principal types of psychological disorders that are encountered within high level sport. The focus of the study is placed on the sex-specific vulnerabilities to particular disorders, and, because the practice of particular sports imposes some unique demands and stressors on athletes, we also explore the occurrence of psychopathology according to the type of sport played.

## Methods

### Ethics

The epidemiological data obtained for this study was collected by retrospectively pooling together results from the yearly psychological evaluations of high level athletes in France, which are mandatory by the law decree of June 16^th^, 2006. This study therefore used a research protocol qualified as non-interventional, in which *“…all acts are performed in a normal manner, without any supplemental or unusual procedure of diagnosis or monitoring.”* (article L1121-1 of the French public health code). According to the law, its approval therefore did not fall under the responsibility of a committee for the protection of persons (CPP). For these reasons, it was not necessary to obtain informed consent from the athletes previously evaluated. All data obtained from collaborating psychologists and physicians was anonymous, and none of the information relayed to the researchers could lead to the identification of any subjects. This study was designed and monitored by two committees: the IRMES (Institut de Recherche bioMédicale et Epidémiologique du Sport) scientific committee and a leading committee dedicated to the study.

### Subjects and data collection

The athletes included in the study were registered on the French High Level or Junior athlete lists. High Level athletes are individuals selected by their sport's national federation for meeting specific performance criteria. Juniors are youth athletes at least 12 years old who are also officially recognized as already delivering strong performances in their age category at the national or international level. A data collection grid was created to standardize the reporting of findings from all athletes' yearly psychological consultations nationwide. This form was not intended for use as a diagnostic tool or a questionnaire, but as a data collection sheet to be filled by the professional after each evaluation. Data was obtained on common psychological disorders, with detail on whether these disorders occurred in the past (over 6 months ago), were current (or recent, within the last 6 months), or either (lifetime occurrence). The following psychopathologies, were included in the report: anxiety disorders (panic attacks, agoraphobia, obsessive-compulsive disorder, generalized anxiety disorder (GAD, duration of at least 6 months) and social phobia); depression (differentiating between major depression and minor episodes by the number of symptoms, at least 2 for minor, and at least 5 for major depression) present over a two week period; eating disorders : anorexia nervosa, bulimia, and eating disorders not otherwise specified (EDNOS); suicidal thoughts and attempts; psychosis; substance abuse and dependence (nutritional supplements, tobacco, cannabis, alcohol, doping agents, psychoactive substances). Diagnoses were based upon DSM-IV or CIM 10 criteria.

Other associated problems were included, such as violent treatment received by the individual, in the verbal, physical, or sexual forms, as well as aggressiveness toward self (auto-aggressiveness) or others (hetero-aggressiveness). Insomnia symptoms (difficulty falling asleep, nocturnal waking, and daytime somnolence) were also assessed.

Socio-environmental factors with the potential of either strengthening or weakening each athlete's psychological well-being were evaluated; the personal and familial environment, athletic environment, academic or professional life, physical health and social life. Each factor was rated on a four point scale. The rating of “1” was considered as a risk factor for the development of psychopathologies, and “2, 3 or 4” as “not a risk factor”. Data was also collected on the athlete's age, sex, height and weight, competition level, type of training center, and the name of the professional performing the evaluation.

This form was sent to all official training centers in France hosting High Level or Junior athletes, and associated medical centers. Information was collected by the physician or psychologist in the training center of each athlete while respecting the rules of confidentiality. The forms were anonymous, and were sent to the IRMES for analysis once completed.

### Data analysis

The presence of psychological disorders (Yes/No) were compared by gender, age (under 18, 18 to 21, and over 21 years of age), type of professional performing the evaluation (physician or psychologist), and geographical region (northwest, northeast, southwest, and southeast of France, and the greater Parisian area) using the Chi-square test, or Fisher's exact test when appropriate. As we focused specifically on the gender differences in psychopathology, a multivariate logistic regression was performed for each disorder adjusting for the potentially confounding factors previously mentioned. The prevalence of each disorder was calculated by dividing the number of individuals affected by the total number of individuals in the group (i.e. all athletes, or men only, women only).

We also compared the presence of the most common psychological disorders encountered in the study sample according to the type of sport played (and by sex) using the Chi-square test and Fisher's exact test. Due to the small number of individuals in many sports in the database, only those including ten or more athletes were included for this analysis. In order to achieve sufficient statistical power, the 36 sports included were grouped into seven categories, similar to those published elsewhere(8); aesthetic, contact/combat, high risk, aiming and fine motor skill, racing, racquet, and team ball sports. We did not have sufficient statistical power to adjust these comparisons on age, type of professional or region.

## Results

2067 athletes' evaluations were returned to IRMES between 2008 and 2009 and included in the analysis. With a total of 16013 High Level and Junior athletes registered on national lists, the sample represents 13% of this population. The proportion of male to female athletes included was representative of the nationwide proportion for high level sport (64.8% compared to 63.9% for men and 35.2% compared to 36.1% for women, respectively. The sample was also representative on the basis of age (18.5±4.9 versus 19.8±5.3 years), ranging from 12 to 35 years old. A similar proportion of athletes belonged to the Junior competition category (55.3 vs. 54.0%), but the High Level category was slightly underrepresented compared to the nationwide proportions (36.3 vs. 46%), likely due, in part, to 9.6% of the evaluations that failed to indicate the athletes' competition category.

The prevalence by sex and occurrence in time (current and lifetime) of each psychological disorder and associated problems included in the data form is presented in Table 1. At the time of their clinical evaluation, 83.1% of athletes did not have any recent or ongoing psychopathology. The 16.9% who did had a mean of 1.4±0.7 disorders over the lifetime, showing several associations between disorders and other problems. Generalized anxiety disorder for instance was associated with major and minor depression, anorexia nervosa, bulimia nervosa and ED-NOS, cannabis abuse and difficulty falling asleep. In turn, major depression was linked to difficulty falling asleep, nocturnal waking, anxiety, being victim to verbal, physical or sexual abuse, alcohol consumption and abuse, and suicide attempts.

**Table 1:** Prevalence of psychological disorders and associated problems (%).

Psychological disorders and associated problems		Current (<6months)				Lifetime (all)			
		All	Men	Women		All	Men	Women	
**At least one psychopathology**		**16.9**	**15.1**	**20.2**	*	**25.1**	**22.1**	**30.8**	***
Anxiety disorders	**At least one anxiety disorder**	**8.6**	**7.1**	**11.3**	**	**12.1**	**10.1**	**15.8**	***
	Anxiété généralisée	6	5.2	7.5	†	8	6.8	10.3	**
	Troubles paniques	1.2	1.1	1.5		2.8	1.9	4.4	*
	Agoraphobie	1	0.8	1.4		1.8	1.3	2.9	*
	Troubles obsessionels et compulsifs	1.6	1.2	2.3	†	1.7	1.5	2.3	
	Phobie sociale	0.8	0.8	0.8		1.3	1.2	1.2	
Depression	**At least one depressive episode**	**3.6**	**2.6**	**4.9**	†	**11.3**	**8.7**	**16.3**	***
	Episode dépressif mineur	3	2.4	4.1	†	9	6.9	13	***
	Episode dépressif majeur	0.7	0.6	0.9		2.6	2	3.5	†
Eating disorders	**At least one eating disorder**	**4.9**	**4**	**6.5**	†	**7.5**	**5.5**	**11.2**	***
	ED-NOS	4.3	3.6	5.9	†	6.2	4.8	9	**
	Anorexia nervosa	0.2	0.2	0.2		1.1	0.5	2.1	**
	Bulimia nervosa	0.4	0.2	0.6		1.4	0.7	2.6	**
Sleep problems (>15 days)	**At least one sleep problem**	**21.5**	**20.2**	**23.9**	†	**26.6**	**24.6**	**30.3**	**
	Difficulty falling asleep	13.4	11.8	16.5	**	17.7	15.6	21.5	**
	Night time waking	7.6	6.1	10.4	***	10.1	8.1	14	***
	Daytime drowsiness	7.5	7.3	7.9		8.3	8	9.3	
Violence received by others	**At least one form of violence**	**3**	**2.8**	**3.3**		**8.8**	**8.4**	**9.5**	
	Verbal violence	2.7	2.6	2.8		7	7	7	
	Physical violence	0.5	0.6	0.4		3.3	3.8	2.3	†
	Sexual violence	0.2	0	0.5		0.8	0.4	1.8	**
Violence inflicted to self or others	Auto-agressiveness	0.8	0.8	0.9		1.8	1.2	2.9	*
	Hetero-agressiveness	2.3	2.9	1.2	**	4	5.1	2	**
Psychosis	Delirious ideas, hallucinations, other	0.2	0.2	0.1		0.4	0.5	0.1	
Suicidal thoughts and attempts	Suicidal thoughts	0.6	0.3	1.2	*	1.9	1	3.8	***
	Suicide attempts	0.3	0.12	0.6		0.5	0.2	1.1	*
Substance consumption, abuse and dependence	**Abuse or dependence-any substance**	**-**	**-**	**-**		**4.1**	**4.2**	**4**	
	Alcohol consumption	20.9	22.8	17.4	***	22.4	24.6	18.5	***
	Tobacco consumption	4.8	4.7	5		6.3	6.3	6.4	
	Cannabis consumption	1.1	1.3	0.9		3.4	3.6	3.1	
	Dietary supplements consumption	11.5	13.2	7.7	**	15.9	18.2	10.9	***
	Illegal perf.-enhancing substances	0.4	0.6	0.2		0.9	1.0	0.6	

Significant difference between men and women;

† , p<0.1,

*, p<0,05,

**, p<0.01,

***, p<0.001 (multivariate analysis, adjusted on age, professional and geographical location).

Regardless of age, type of professional or geographical location, women were 1.3 times more likely to be diagnosed with at least one psychopathology than men. While a similar proportion of men and women had only one disorder in their lifetime, more than twice as many women were diagnosed with 2 or more disorders than men (5.8 vs. 2.4% currently, and 13.0 vs. 5.6% over the lifetime). Younger age also impacted the prevalence of current psychopathology, with 15.1% of athletes aged 17 years or under showing at least one disorder, against 13.1% of athletes between 18 and 21 years old and 10.4% of athletes 22 years and over (p = 0.04).

Generalized anxiety was the most prevalent disorder found in men and women. A significant gender difference appeared in anxious pathologies, showing women as 56% more likely to have suffered from any anxiety disorder over their lifetime than men. A large majority (82.4%) of men diagnosed with generalized anxiety disorder had no other anxious disorders, while women were concomitantly diagnosed with OCD, agoraphobia or panic disorder significantly more often (44.3% of GAD cases).

Recent or ongoing depression was encountered in 3.6% of athletes, with 87% of cases classified as minor depression. Only 7 men and 6 women were diagnosed with recent or ongoing major depression. Compared to men, nearly twice as many women were found to have experienced minor and/or major episodes of depression over the lifetime. Age was also associated with ongoing major depression, affecting 2.2% of athletes over 21 years of age, 0.8% in 18 to 21 year olds, and 0.3% in athletes under 18 (p = 0.02).

Ongoing eating disorders were found in 4.9% of athletes, the vast majority classified as eating disorders not otherwise specified (ED-NOS). Only 3 athletes were diagnosed with ongoing anorexia nervosa, and 7 with bulimia nervosa. Women were significantly more likely than men to have or have had anorexia, bulimia, or an ED-NOS in their lifetime.

Relative to the total number of women and men diagnosed with generalized anxiety, depression, or an eating disorder, the occurrence of all 3 of these disorders over the lifetime was identified significantly more often in women than men (8.1% vs. 0.5% of cases, respectively)

Ongoing sleep problems were experienced by 21.5% of the athletes at the time of their evaluations, difficulty falling asleep being the most common. While difficulty falling asleep and nocturnal waking occurred more often in women, the prevalence of daytime somnolence was significantly greater in younger athletes (10.5% in 17 year-olds and less, versus 8.2% in 18 to 21 year olds and 3.3% in 22 year olds and up, respectively).

3% of athletes were found to be or to have been subjected to some form of violence, and no differences were found according to sex. Verbal harassment or aggressions was the most common type of violence that athletes experienced, followed by physical, then sexual forms of violence. Younger age was linked to increased exposure to verbal abuse, as it involved 7.4% of those aged 17 years and under but only 4.3% of the older athletes. 1.9% of athletes had had suicidal thoughts at some point in their life, women more often than men. 7 women and 3 men were found to have attempted suicide at some point. As expected, these 10 individuals had significantly more socio environmental risk factors and had several other issues; 8 had been depressed, 8 had been violence victims, 7 had an anxiety disorder, and 5 an eating disorder.

Alcohol was the substance whose consumption was the most reported, with 20.9% of athletes admitting to consuming alcohol at least rarely or occasionally. Men consumed alcohol and dietary supplements more often than women, and while greater age was associated with increased alcohol (p = 0.04) and tobacco (p = 0.0001) consumption, athletes aged between 18 and 21 years appeared as the most frequent cannabis consumers.

The type of professional performing the psychological evaluations caused a significant bias on the prevalence of psychopathology identified. Physicians detected one or more psychological disorders over the lifetime in 23% of women and 14% of men, compared to 34% and 28% of those evaluated by psychologists. Psychologists identified more “stand alone” disorders, while physicians and psychologists detected similar proportions of individuals having had 2 or more disorders. Since a slightly higher proportion of women were evaluated by psychologists than men (63.8 and 56.4% respectively), all gender comparisons were adjusted on the type of professional.

Table 2 shows the association between the presence of each socio-environmental risk factor and the presence of psychological problems over the lifetime. The presence of any socio-environmental risk factor was strongly associated with the occurrence of a psychological disorder over the lifetime in both sexes. Each risk factor was found more often in women. Further, among the athletes exposed to any of these risk factors, women were significantly more likely than men to have developed at least one disorder in their lifetime.

**Table 2:** Prevalence of at least one psychopathology over the lifetime in athletes with and without socio-environmental risk factors.

Socio-environmental factors	Risk factor?	Athletes, %	Athletes within each level of risk with ≥1 psychologicaldisorder , %				
			Men		Women		
Personal and family life	**At risk**	**2.4**	**46**	‡‡	**84.2**	‡‡	**
	Not at risk	98.0	16.5		26.9		***
Sport environment	**At risk**	**1.6**	**62.5**	‡‡	**73.3**	‡‡	
	Not at risk	98.5	20.6		27.6		***
Scholastic and professional life	**At risk**	**3.6**	**43.1**	‡‡	**63.2**	‡‡	
	Not at risk	96.6	23.1		27.7		*
Physical health	**At risk**	**3.4**	**48.8**	‡‡	**76.9**	‡‡	*
	Not at risk	96.7	20.3		26.7		**
Social life	**At risk**	**0.8**	**50**	‡	**66.7**	‡	
	Not at risk	99.2	21		28.1		***

Significant difference in the prevalence of psychopathology between athletes with or without the risk factor:

‡ p<0,05,

‡‡ , p<0.001.

Significant difference between men and women:

*, p<0.05;

**, p<0.01,

***, p<0.001.

**Figure 1:**
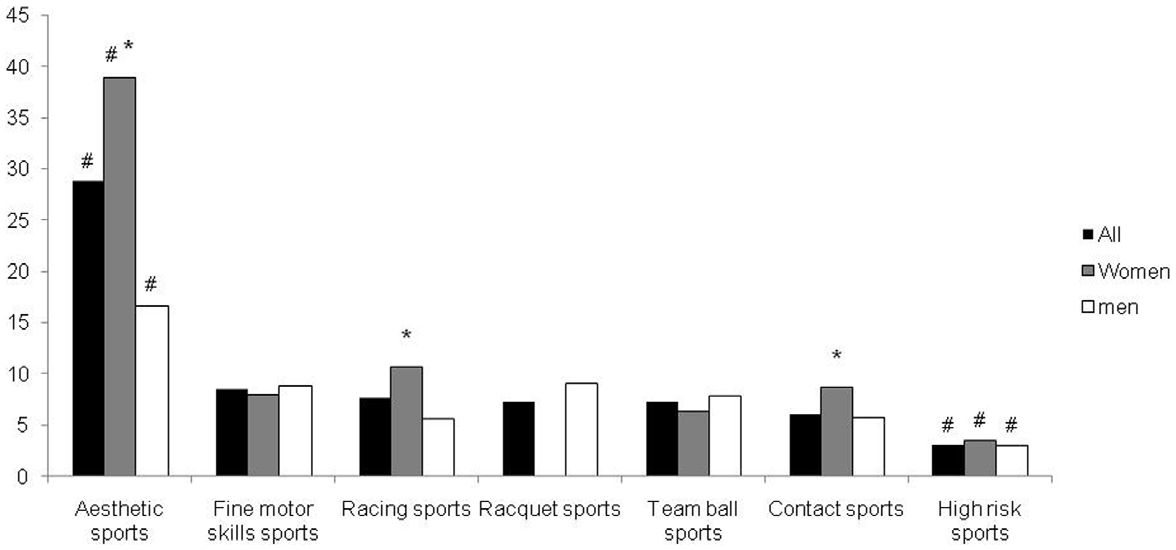
Lifetime prevalence (%) of generalized anxiety by type of sport played. #, significant difference with all other sports (p<0.05). *, significant difference between men and women (p<0.05).

The occurrence of lifetime generalized anxiety disorder (GAD) according to the type of sport played is presented in Figure 1. Significantly higher rates of GAD were found in aesthetic sports (38.9 vs. 10.3% for women in all other sports, and 16.7 vs. 6.8% for men in all other sports, respectively). The gender difference in the prevalence of GAD remained significant within aesthetic sports. High risk sports had the lowest prevalence of GAD for both women and men (3.5 and 3.0% respectively).

**Figure 2:**
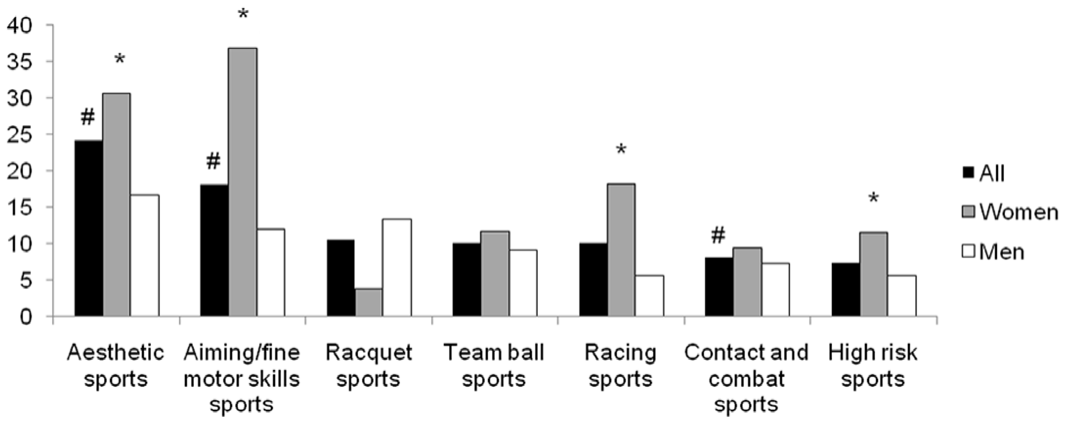
Lifetime prevalence (%) of minor or major depression according to the type of sport practiced. #, significant difference with all other sports (p<0.05). *, significant difference between men and women (p<0.05).

**Figure 3:**
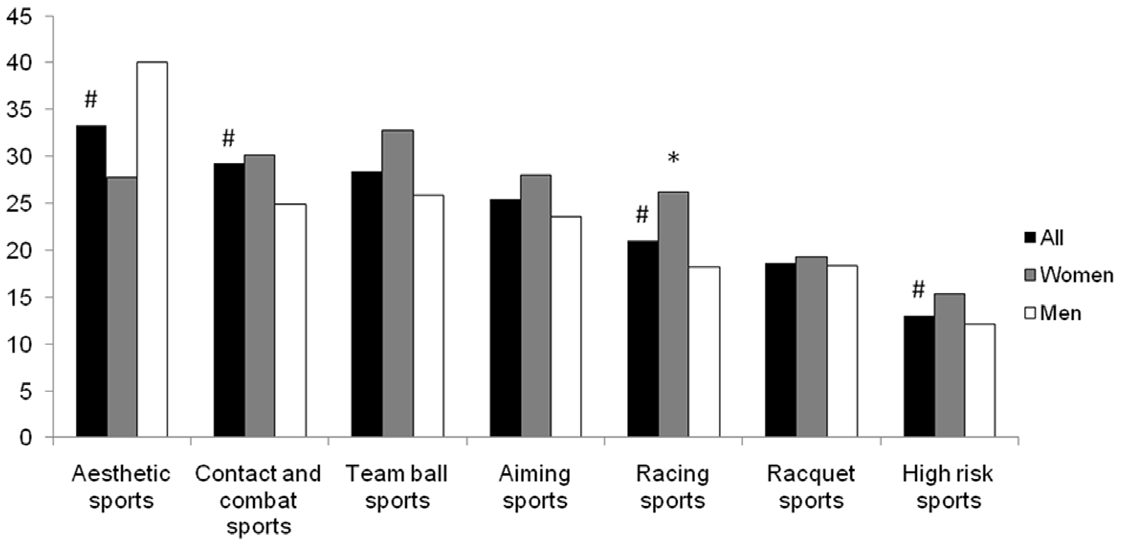
Lifetime prevalence (%) of all sleep problems according to type of sport played. #, significant difference from all other sports (p<0.05). *, significant difference between men and women (p<0.05).

The prevalence of depression and sleep problems according to the type of sport practiced followed the trend observed with GAD (Figures 2 and 3). The lifetime occurrence of at least one period of depression was highest in aesthetic sports (24.2%), followed by aiming and fine motor skills sports (18.2%). The rate of depression was significantly lower in team ball sports (8.1%), and with 7.4%, high risk sports showed the lowest prevalence of depression, but did not reach significance (p = 0.19 according to the Chi square test) due to the low numbers of athletes. Sleep problems were also more prevalent in aesthetic sports (33.3%), while athletes in high risk sports had significantly less sleep issues than the rest.

The lifetime occurrence of any eating disorder showed different sport-specific trends in men and women. The highest rates of eating disorders were found in women involved in racing sports and fine motor skills sports, while those playing team ball sports had the lowest occurrence of such problems (14, 14 and 5.8% respectively). For male athletes however, participation in combat and contact sports showed the highest prevalence of eating disorders (7.3%).

## Discussion

This study brings forth two important themes of psychology in elite sport. First, the gender-based differences in psychopathology demonstrated here agree with findings from other epidemiological studies performed on the general population [14]–[17], [20], [21]. Elite female athletes are more likely to be diagnosed with a psychological problem than men, and appear more susceptible to difficulties encountered in their environment than their male counterparts. Secondly, important variations in these occurrences according to the type of sport practiced are brought forth, conveying that the demands and pressures associated with the practice of a particular sport may act as one of the significant socio-environmental risk factors which, if combined with a particular personality and genetic predisposition, could facilitate the development of some disorders.

GAD is the principal psychological issue encountered in both genders. Characterized by excessive, difficult to control worrying that disrupts daily functioning, this disorder usually becomes full blown in late adolescence or early adulthood, showing both cognitive and physical symptoms. In the general population, anxiety disorders have a high comorbidity with other psychopathologies, such as major depression, eating disorders, substance abuse and drug addiction [15], and an array of long-term psycho-physiological consequences that are often closely intertwined. Such associations between anxiety and other disorders are demonstrated here in elite athletes. The 3∶2 women to men ratio found for GAD in this study resembles that usually reported in large population studies [14], [22]. Researchers have proposed explanations for this female predominance in anxious disorders at the genetic, physiological, and socio-environmental levels [23]. From a hormonal perspective, one proposed mechanism involves the gene expression of corticotropin releasing factor (CRF), a neurohormone which initiates the autonomic and cognitive response to stress, and which is directly regulated by estrogen [24]. CRF acts on the locus ceruleus (LC), a brain center known to regulate emotional arousal via the secretion of norepinephrine. The excessive activity of CRF and LC systems is considered a major component underlying the development of stress related disorders like depression and anxiety. Bangasser et al. [25] demonstrated that the activation of the CRF and LC-norepinephrine systems occurred in response to subthreshold stressors in female compared to male rats, reinforcing the concept of sexual dimorphism in physiological stress reactivity.

From the psychosocial perspective, it is thought that women's biological tendencies for increased worrying are often strongly reinforced by gender-norms socialization throughout their development [25]. Regardless of their relative influence, the predominance of women suffering from anxious or affective disorders is most likely due to a combination of these fundamental differences at the biological and psychosocial level.

Finding generalized anxiety as the most prevalent disorder in both male and female athletes does not come as a surprise. The age range of athletes (adolescence and early adulthood) corresponds to that during which the highest rates of anxiety are usually found; Leray et al. [15] found that in the French population, about 12.8% of individuals had GAD, with the highest rates found in 18 to 34 year-olds (14.3%). Just as unsatisfactory school performance is often a source of anxiety in adolescents [26], it is easily conceivable that the pressure to excel within their sport could present a risk factor in high level athletes; especially if a large discrepancy exists between the desired or imposed goals and actual performance, and thus becomes a source of excessive worry. Yet despite the pressures inherent to high level sport, the overall prevalence of GAD in athletes appears no higher than in the population.

Our results support the notion that the particular constraints associated with specific types of sports might play a role in the development of GAD. The greatest prevalence of this disorder is found here in athletes practicing aesthetic sports; gymnastics, synchronized swimming and figure skating. This raises an interesting point regarding the possible psychological repercussions of being so highly invested in a sport in which success is heavily dependent upon judgement by others -jury members, coaches, or even teammates - and the pressure to deliver a ‘perfect’ performance is therefore tremendous [27]. At the highest levels of competition, differences in the quality of each performance can be extremely small, and obtaining a medal or finishing last can be decided by fractions of a point. Although trained to score objectively, judges can be inconsistent or affected by a number of factors, including the nationality of the athlete, political inclinations, or even the public effect, to name a few [28], [29]. In a study by Kerr et al. [30] elite gymnasts have expressed the feeling of “lacking control, not only over the ultimate outcome of their performances, but also many other aspects of their careers”. The sense of powerlessness in important aspects of life is well documented as one of the triggering factors for anxiety disorders and depression [30]–[32]. The tremendous competition-related state anxiety in judged aesthetic sports should certainly not be confused with pathological anxiety. However, it has been speculated that one can evolve into the other in athletes who show “negative perfectionism”, in that their self esteem is largely based upon competence aspects and success in their endeavours [28], [33]. We speculate that these characteristics, combined with the specific pressures of judged sports, may help to explain the high occurrence rate of GAD in these athletes.

On the other end of the spectrum, high risk sports, which include sliding sports, aerial sports and motor sports, show the lowest rates of GAD. These sports all share the very high risk of lethal accidents inherent to each performance [34], [35]. Commonly referred to as “thrill seekers”, individuals such as high risk sports participants may need higher levels of stimulation in order to compensate for a chronic deficit in basal arousal [36], [37]. In addition, it could be argued that these elite high risk sports athletes may have a better propensity to cope with stress or to deal with fearful situations, sometimes through an “optimistic bias” [38]. In light of this, the low GAD rates found in our study could support the interpretation that individuals with anxious predispositions, excessive worriers unable to manage fear and stress well, would be very unlikely to practice such dangerous sports.

If the prevalence of GAD resembles that of the population, the rate of major depression, the most widely diagnosed and costliest mental health problem encountered in many countries, is by comparison very low within the high level athlete population. In spite of this, the 2∶1 women to men ratio consistently reported in population studies [14], [22] was preserved in the athlete population. As for anxiety disorders, this phenomenon is partially affected by the manner in which men and women express their symptoms, and women's greater willingness to talk about them [39]; the social stigma of weakness associated with depression also makes men less likely to seek help from a professional than women.

In addition to these social differences, the female preponderance in depression also appears to share some of the same biological origins as anxiety disorders, and is thought to be a secondary effect to women's predominance in anxiety disorders. Parker et al. [16] have reported that both female sex and preceding GAD contribute significantly to the development of major depression, with GAD being the stronger contributor of the two.

In a large study, Alonso et al. [14] found a lifetime prevalence of major depression of 12.8%, and a 12-month prevalence of 3.9%, with the greatest rates found in 18 to 24 year olds. Our results indicate that less than 1% of athletes were suffering from major depression within the 6 months leading up to their evaluation, and 2.6% over the lifetime. Even though we may only loosely compare the prevalence of depression across population studies, the tremendous difference between that found within our sample population and those of the greater population is noteworthy and encouraging for high level sport.

The literature already strongly supports the anti-depressive benefits of physical exercise [1], and our results show that lower depression rates are also found within high level sport. Despite the increased pressures and constraints exerted upon elite athletes relative to those in recreational sports, the former could potentially be more resilient to stressors that would otherwise trigger depression symptoms in many individuals. Elite athletes are characterized by their peers as strongly optimistic and able to adapt and even thrive under pressure [13]. As researchers are determining which genes may confer physiological or physical advantages to elite athletes [11], [12], the same concept could be studied for psychological resilience in these individuals. Gene polymorphisms have been identified which confer either resilience or vulnerability to stress, fear, and in turn, the development of depression [40]–[42]. While we have not measured resilience or adaptability, the low prevalence of major depression in this group incites us to consider that psychological resilience could be part of many elite athletes' genetic attributes.

The trends in lifetime depression by type of sport practiced followed, as expected, those seen for GAD, since the two disorders were closely associated in men and women. The higher prevalence of depression in aesthetic sports athletes reinforces the idea of a greater “psychological toll” within these jury-based disciplines.

The majority (86%) of eating disorders encountered in this study were classified as EDNOS. The percentages of athletes with anorexia or bulimia nervosa, are similar to those obtained on a large sample of the European population aged 18 to 29 years [17]. However, the 2∶1 women to men ratio was smaller in this study than those reported in the population (between 6∶1 and 10∶1) [8], [17]. This could be due in part to a higher number of male athletes displaying minor disordered eating in sports with weight category constraints.

As expected, the prevalence of disordered eating varied widely according to the type of sport practiced, likely reflecting the constraints inherent to each type of discipline. While women in racing sports (particularly track and field, a weight-bearing sport) and aiming/fine motor skill sports were more affected by disordered eating than in other sports, women in team ball sports, racquet sports and aesthetic sports fared the best in that area. While much concern exists over disordered eating in this population [7], [8], [43]–[45], more recent research by Klinkowski et al. [46] found that despite their often extremely thin physique, rhythmic gymnasts did not share the psychopathological features of anorexic patients.

For men, the practice of contact and combat sports was associated with the highest prevalence of disordered eating. The literature provides ample documentation on inappropriate dieting behaviors associated with weight class sports [47]–[50]. While many of these dieting practices in weight-class sports are unhealthy and should be discouraged, it is also important to recognize that many high level weight-class athletes do not suffer from a true, psychopathological eating disorder. Once the performance-specific pressure to drop weight is removed, at the end of a season for instance, most athletes regain normal dietary habits, and do not show the psychological distress and preoccupation with weight indicative of a psychopathology [47], [48].

An important goal behind the nationwide implementation of a psychological follow-up for high-level athletes consisted of identifying socio-environmental risk and protection factors that could influence the psychological well-being of each individual. The strong link that emerged between these factors and the presence of psychopathology strengthens the concept that particular attention should be paid to the well-being of athletes by ensuring that adequate social or professional support remains accessible.

Our study encountered a few of the limitations typically associated with epidemiological studies. The professional bias induced by the fact that 61% of athletes were seen by psychologists and 38% by physicians probably resulted in lower rates of psychopathology than would have been reported by psychologists only. Even though psychologists identified more athletes with one disorder, physicians found as many individuals with 2 or more disorders as psychologists. This could be partially due to differing sensitivities for recognizing disorders that may difficult to diagnose. For instance, among eating disorders, psychologists diagnosed more EDNOS, minor EDs, than physicians, but physicians identified anorexia and bulimia as often as psychologists. In the realm of high level sport, the fear of stigmatization for psychological problems could be especially strong; elite athletes work extremely hard to excel, and might therefore be particularly unwilling to disclose anything that could reveal emotional or mental fragility. Despite these limitations, our study had the merit of being based on in-person consultations by health professionals.

This study is the first to examine the prevalence of the major types of psychopathology over a nationwide sample of high level athletes; the results obtained are encouraging, as they assert the psychological balance of this population despite the stress imposed by athletic careers. The expected sex-based differences in the prevalence of various disorders are demonstrated, showing that the sex-specific trends in psychopathology resemble those of the population at large. The practice of a sport at the high level, in itself, does not appear psychopathogenic, since the prevalence of psychopathology identified is no higher than in the general population. Rather, it is the presence of very particular stressors, such as problems in the athletes' social, personal and sporting environment that is associated with psychopathology. Psychological issues and the stressors from which they stem should be addressed early, in order to help avoid the development of a full-blown disorder and its potential consequences on the athletes' health and career.

## Acknowledgments

We would like to thank all of the psychologists and physicians who collaborated with the IRMES since 2007 and helped us to gather data from the psychological evaluations of high level athletes: E. Rosnet, E. Mangon, I. Inchauspé, J.M. Sene, C. Chalopin, O. Laigneau, P. Mouret, S. Ruffio-Thery, J. Girardin, I. Chevrier, J. Rougier, A. Favre, L. Desmurget, L. Hennebaut, S. Gimenez, M. Bonnier, C. Doé de Maindreville, B. Brunet, C. Quignon-Fleuret, M. Salmi, J.C. Guibert, M. Guinaudeau, E. Chedhomme, K. Reperant, M. Paquinet, Y. Guillot, A. Merllie, N. Crepin, B. Blanchard, E. Volle, D. Vassal, D. Gutierrez, M.P. Mazière, F. Pelletier, B. Sesboué, F. Granet, Y. Hervouet des Forges, D. Rousseau, O. Coste, A. Frey and the many other health professionals who participated. We would also like to thank the INSEP teams for their full support.
